# Anxiolytic effects of fluoxetine and nicotine exposure on exploratory behavior in zebrafish

**DOI:** 10.7717/peerj.2352

**Published:** 2016-08-24

**Authors:** Matthew L. Singer, Kris Oreschak, Zachariah Rhinehart, Barrie D. Robison

**Affiliations:** 1 Department of Biological Sciences, University of Idaho, Moscow, ID, United States; 2 Skaggs School of Pharmacy and Pharmaceutical Sciences, University of Colorado Anschutz Medical Campus, Aurora, CO, United States

**Keywords:** Behavior, Anxiety, Fluoxetine, Nicotine

## Abstract

Zebrafish (*Danio rerio*) have emerged as a popular model for studying the pharmacology and behavior of anxiety. While there have been numerous studies documenting the anxiolytic and anxiogenic effects of common drugs in zebrafish, many do not report or test for behavioral differences between the sexes. Previous studies have indicated that males and females differ in their baseline level of anxiety. In this study, we test for a sex interaction with fluoxetine and nicotine. We exposed fish to system water (control), 10 mg/L fluoxetine, or 1 mg/L nicotine for three minutes prior to being subjected to four minutes in an open-field drop test. Video recordings were tracked using ProAnalyst. Fish from both drug treatments reduced swimming speed, increased vertical position, and increased use of the top half of the open field when compared with the control, though fluoxetine had a larger effect on depth related behaviors while nicotine mostly affected swimming speed. A significant sex effect was observed where females swam at a slower and more constant speed than males, however neither drug produced a sex-dependent response.

## Introduction

The zebrafish (*Danio rerio*) is a popular research model for studying pharmacology (summarized in Barros et al., 2008; Langheinrich, 2003) and behavior (Gerlai, 2015), particularly with regard to stress and anxiety. The zebrafish provides a vertebrate model that breeds rapidly, is easy to maintain in large numbers, and can be administered drugs through immersion. Zebrafish also share many of the same neurotransmitters (Shin & Fishman, 2002) and stress pathways as humans, utilizing cortisol rather than corticosteroids as used by rats and mice (Barcellos et al., 2007). These features have facilitated zebrafish studies on addiction (Mathur & Guo, 2010), learning (Sison & Gerlai, 2010), social behavior (Buske & Gerlai, 2014; Gerlai, 2014) and anxiety behaviors (Mathur & Guo, 2010; Maximino et al., 2010).

Anxiety-related behaviors are known to vary by sex in zebrafish and other model organisms, and these differences may be explained by gonadal hormones (Zimmerberg & Farley, 1993; Palanza, 2001). Male and female rats differ in their time spent in the center of an open field and a plus maze, though the nature of these differences are also dependent on the strain observed (Mehta, Wang & Redei, 2013). In zebrafish, females tend to be less anxious, or more bold, than males when measuring location preferences in the presence of a human observer (Benner et al., 2010; Oswald et al., 2013).

Drugs are used to manipulate anxiety and related disorders in humans and are also utilized as a tool for understanding behavior. Fluoxetine, for example, is a drug used to treat depression and anxiety. It works by blocking the reuptake of serotonin in the brain (Beasley, Masica & Potvin, 1992). Serotonin and its transporters have been associated with anxiety (Graeff, Viana & Mora, 1997; Lesch et al., 1996). Nicotine is naturally found in tobacco products and binds to nicotinic cholinergic receptors (nAChRs) to release dopamine (Benowitz, Hukkanen & Jacob, 2009). The result is an anxiolytic response (Picciotto, Brunzell & Caldarone, 2002).

Observations of male and female differences in anxiety-related behavior have led us to ask whether the effects of anxiolytic substances also differ by sex. There is evidence that the effectiveness of anxiolytic drugs may vary with sex in mammals. Differential responses have been observed in humans utilizing Sertraline, a selective serotonin reuptake inhibitor (SSRI) where females showed an enhanced response compared to males (Kornstein et al., 2000). Sex-specific differences were observed in the effectiveness of the SSRI Fluoxetine in humans (Martényi et al., 2001), and studies utilizing rats (Mitic et al., 2013; Leuner, Mendolia-Loffredo & Shors, 2004; Lifschytz et al., 2006) and mice (Monleón et al., 2002; Hodes et al., 2010) have shown a discrepancy between the sexes in both the physiological and behavioral responses to this drug where efficacy tends to be greater in females than in males. Evidence in rats also suggest that nicotine’s effects on stress and anxiety may also differ between the sexes with males exhibiting a greater anxiolytic effect (Faraday, O’Donoghue & Grunberg, 1999). This is important from a pharmacological standpoint in that effective doses may differ between males and females. On a broader level, studies utilizing a single sex, or ignoring the effect of sex altogether ought not to be used to draw broad conclusions about the effects of that drug.

While zebrafish are becoming a model for pharmacological research, literature describing sex-dependent effects of anxiolytic drugs in this system are lacking. In this experiment, we test the hypothesis that zebrafish exhibit sex-dependent responses to fluoxetine and nicotine. These substances were chosen because they have known anxiolytic effects across a wide variety of model systems including humans (Gilbert, 1979; Griffin & Mellon, 1999), rats (Cohen et al., 2009; Zhang et al., 2000) and zebrafish (Bencan & Levin, 2008; Bencan, Sledge & Levin, 2009; Cachat et al., 2010; Levin, Bencan & Cerutti, 2007), and while sex-specific effects have been observed in mammals, studies in zebrafish utilizing these substances largely ignore the effects of sex.

## Methods

### Subjects

Experimental fish were bred from adult ScientificHatcheries strain (Huntingdon, CA) that has been maintained in our facility.Water in our Aquaneering Inc. (San Diego, CA) system was constantly circulating and kept at a temperature of 28.5°C on a 14 hour light:10 hour dark cycle. The fish were fed a diet of brine shrimp twice and flake food (Tetramin) once for a total of three daily feedings. At the time of data collection, the fish were four months old and housed in three-liter tanks in groups of five to achieve maximal growth rates. Though zebrafish stocked at this density are known to develop social hierarchies that can influence stress and behavior (Pavlidis et al., 2013), we randomly assigned individuals to a drug treatment group such that these effects should be equally distributed across treatments. All aspects of this study were approved by the University of Idaho’s Animal Care and Use Committee under protocol 2014-14.

### Dosing

Fluoxetine (generic (Teva Pharmaceuticals) from Wal Mart) and nicotine (Sigma Aldrich) treatments were administered at concentrations of 10 mg/L for the fluoxetine and 1 mg/L for the nicotine. These doses vary from standard doses in the zebrafish literature. Fluoxetine is often given at concentrations up to 100 μg/L, but administered chronically over a two-week period (Egan et al., 2009). We used a higher dose than the chronic concentrations reported in the literature; however, it is important to note that this choice could yield non-target effects due to higher concentrations. Nicotine is often administered as a ditartrate salt at concentrations up to 100 mg/L (Levin, Bencan & Cerutti, 2007). We used pure nicotine and were unsure at the time of the experiment how the two forms compared with each other. We chose our dose based on the LD50 concentration (4 mg/L) to avoid lethal effects on our subjects. Each drug was dissolved in system water to make a working solution each morning of administration. A third treatment of only system water served as a control. Fish were netted from their home tank and immediately placed into a beaker containing 100 mL of one of the three treatments. After three minutes of exposure to the drug dose, the fish were transferred to an open field test tank filled with untreated system water for behavioral recording. Dosing and behavioral observations were made on one fish at a time and the treatment type and order were randomized across individuals.

### Behavior assay & video tracking

The fish were placed in a rectangular tank with interior dimensions measuring 25 cm wide, 12 cm high (from water level to bottom), and 6 cm thick (front to back). The volume of water in the tank was approximately 2 L. Each fish was filmed for four minutes (240 s) at 25 frames/second beginning from the time that the subject entered the water. The camera and operator were hidden behind a blind during the recorded observation time. The tank was backlit with an opaque diffuser for the purposes of creating a silhouetted object for motion tracking. After the four-minute period, the fish was netted out of the test tank, placed into its own individual 1.5 L housing and returned to the main system. Observations were recorded over three days between the hours of 10:00 am and 2:00 pm. After all subjects had been recorded, weight and standard length measurements were obtained by first anesthetizing the individual in MS-222 solution and blotting excess water with a paper towel. At this time, we also recorded the sex of the individual using visual cues: larger, rounded abdomen and dull fins for females, smaller and leaner abdomen and bright yellow fins for males.

Videos were digitally tracked using ProAnalyst^^®^^ (Xcitex, Cambridge, MA). Tracking began with the frame in which the fish hit the surface of the water, and proceeded to the end of the video. The tracking data were imported into R for cleaning and processing. Each track was truncated to exclude the first five seconds during which the fish would sink, but remain otherwise motionless, as it recovered from the initial shock of being released from the net. Tracks were then standardized to 4 min, or 6,000 frames. We computed velocity from the x–y data points. Since the tracking software did not always track the exact same position on the fish, velocity was estimated using the change in coordinates between two frames before and two frames after the focal frame. This algorithm sufficiently smoothed the speed data while retaining detail at small time intervals.

### Analysis

#### Freezing

Freezing time was defined as the time a subject spent motionless on the bottom of the tank. We defined motionless as maintaining a velocity of less than .01 cm/frame for more than 20 consecutive frames. Any short bursts of motion flanked by considerable freezing times were verified in the video to be true motion. If a time period of activity was less than 40 frames, it was re-categorized as part of the freezing time as this motion is likely an artifact of the automated tracking. The freezing time was then calculated by counting the total number of frames marked as frozen. We also characterized freezing behavior as a binary ‘yes’ or ‘no’ response as the propensity to show any freezing behavior can be considered an independent response from duration of freezing.

#### Speed

We computed the average speed for each individual using only the active (non-frozen) data points from the swim tracks. Freezing behaviors can cause a high degree of correlation with average swimming behaviors such as speed and depth use. Since we analyzed freezing behavior separately, we chose to analyze the effects of anxiolytic drugs on velocity during active swimming only. We predicted that anxious individuals would swim slower on average than less anxious individuals (Gerlai, Fernandes & Pereira, 2009). In addition, we computed the variance in velocity for the active data points. The variance represents the consistency in swim speed within an individual. Less anxious individuals should display more consistency in velocity than more anxious individuals due to erratic behavior (Gerlai, Fernandes & Pereira, 2009).

#### Depth

Depth was measured by the y-coordinate position in the swim track. We aligned the y origin with the water’s surface, and measured depth as increasing negatively toward the bottom of the tank. As with velocity, depth variables were calculated using only the active points in the tracks. We analyzed both the mean and variance (consistency) of depth. We predicted that anxious individuals should spend more time near the bottom of the tank and should have a lower variance in depth (Levin, Bencan & Cerutti, 2007; Oswald, Singer & Robison, 2013). Conversely, we predicted that less anxious individuals will position themselves higher in the water on average and spend more time exploring the entire tank, resulting in a larger variance in depth usage. We also quantified at the number of times an individual entered the top half of the tank from the bottom half. Such behavior may be indicative of anxiety, as anxious individuals tend to enter the top half less often than less anxious individuals (Egan et al., 2009). We also expected that anxious individuals would spend a smaller proportion of active swimming time in the top half, and that they would exhibit a longer latency to enter the top from the beginning of the trial (Egan et al., 2009). The threshold between the top and bottom halves was defined at −6 cm.

#### Horizontal place preference

The width of the tank was divided into three equal sections and the proportion of time in the middle section calculated to differentiate preference to be located in the center versus the edge of the test environment. While we had clear expectations for location preference with respect to depth, it was unclear at the time of analysis whether the middle or the edges represent a “safe” zone with respect to horizontal preference. Experiments with rodents have found that stressed individuals prefer the edges of their arenas (thigmotaxis), but that this behavior is analogous to stressed fish preferring the bottom (Levin, Bencan & Cerutti, 2007).

#### Statistical analysis

We began with a MANOVA on all continuous variables where all individuals could be included. We applied transformations where they were required to conform to the assumptions of normality in the residuals (see Results for transformations). The initial model included the effects of weight as a covariate, sex, drug treatment, and the sex by drug interaction. No significant effect of weight was observed, and there was no improvement to the model by keeping the term, so we excluded weight from all subsequent analyses. We performed individual ANOVAs on each of the continuous variables. Since freezing occurrence is a binary response, it was analyzed using a logistic GLM to estimate and compare the probability that an individual will freeze based on a given treatment group. In order to accurately assess freezing time, only individuals that froze were used (*N* = 52). All tests were performed with a significance threshold of *α* = 0.05. When a significant effect of drug treatment was detected, we performed pairwise T-tests among the three treatments with a Tukey correction.

## Results

We recorded observations from 90 individuals divided equally and randomly among the 3 treatments (*n* = 30 per treatment). Due to complications with the filming, observations on three of the individuals had to be removed leaving us with final sample size of 87 individuals broken down by treatment and sex as follows: 29 in the control treatment (17 females and 12 males), 30 in the fluoxetine treatment (16 females and 14 males), and 28 in the nicotine treatment (14 females and 14 males).

### Multivariate

The full model Type-II MANOVA included the effects of weight, sex, drug treatment, and the sex by drug interaction on average depth, variance of depth, average speed, variance of speed, percent of time spent in the top half, number of crosses into the top half, latency to enter the top half, and proportion of time spent in the middle third horizontally (i.e., away from the edges). There was a non-significant effect of sex (Λ = 0.17896, *F*_8,73_ = 1.9889, *p* = 0.05974) and a significant effect of drug treatment (Λ = 0.56646, *F*_16,148_ = 3.6551, *p* = 0.00001305) on behavior, but no significant interaction. There was no significant effect of weight as a co-variate, and including weight in the model showed no improvement over removing it (Λ = 0.95793, *F*_5,76_ = 0.66755, *p* = 0.6492). With the reduced model, we observed a significant effect of sex (Λ = 0.22404, *F*_8,74=2.6707_, *p* = 0.01237) and drug treatment (Λ = 0.56659, *F*_16,150_ = 3.7057, *p* = 0.00001014). Therefore, for all subsequent analyses we considered only the effects of sex, drug treatment, and the interaction term.

### Individual components of behavior

We observed no significant interactions between sex and drug treatment in any of the individual behavior components (see Table 1), consistent with the results of the MANOVA above. All components indicated a significant effect of drug treatment (*p* < 0.05) except for freezing occurrence and freezing duration. The subsequent descriptions describe the results of the post-hoc pairwise comparisons of the drug treatments using the least-squared means and Tukey adjusted *p*-values based on 3 tests. We also observed a significant effect of sex with regard to average swimming speed (*F*_1,81_ = 10.7178, *p* = 0.001562) and consistency (variance) of swimming speed (*F*_1,81_ = 13.9196, *p* = 0.0003528). Males were on average faster than females, but also exhibited less consistency in their swimming speeds. These were the only instances in which the sexes differed in their behavior.

**Table 1:** Table of *P*-values summarizing results. **Bold** items are considered to show significant differences among treatment groups (*α* = 0.05). *P*-values for the Fluoxetine and Nicotine columns represent pairwise comparisons with the control and are adjusted using the Tukey method for 3 comparisons.

	Sex	Drug	Interaction	Fluoxetine	Nicotine
Freezing Time	0.17	0.26	0.76	0.99	0.33
Average Speed	**0.00**	**0.01**	0.63	0.13	**0.00**
Variance Speed	**0.00**	**0.02**	0.47	0.57	**0.02**
Average Depth	0.98	**0.00**	0.94	**0.00**	**0.04**
Variance Depth	0.62	**0.01**	0.91	**0.01**	0.19
Proportion in Top	0.86	**0.00**	0.72	**0.00**	0.22
Crosses to Top	0.57	**0.00**	0.89	**0.00**	0.45
Latency to Top	0.64	**0.00**	0.89	**0.03**	**0.00**
Proportion in Center	0.19	**0.00**	0.36	**0.00**	0.99

#### Freezing behavior

**Figure 1:**
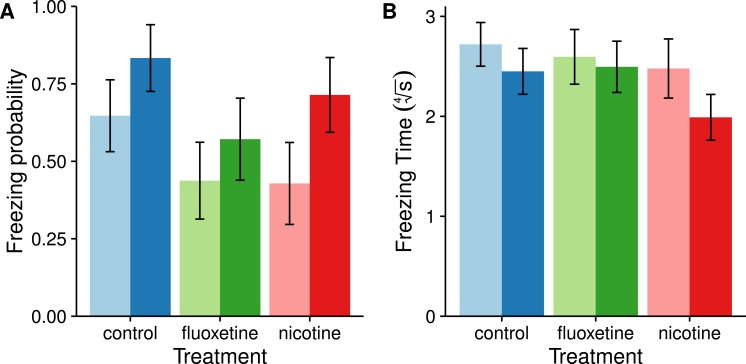
Freezing behaviors (motionless at the bottom of the tank) appear not to be affected by exposure to fluoxetine or nicotine. These graphs show the probability of freezing ± SE. (A) and the mean time spent frozen ± SE (B) for both sexes in each drug treatment group. Females are represented as light bars and males as dark bars. The freezing probability was calculated from a logistic GLM and transformed back into probabilities for this figure using the ‘lsmeans’ package in R. Freezing time was transformed using a fourth root in order to meet the assumptions of normality in the ANOVA.

Freezing behavior is a commonly observed anxiety related behavior in zebrafish (Egan et al., 2009). Of the 87 individuals observed, 52 exhibited freezing behavior. Though males tend to be more likely to freeze than females on average, this difference was not statistically significant (*χ*^2^ = 3.7866, *p* = 0.05167). We also failed to observe a significant effect of drug treatment on freezing occurrence (*χ*^2^ = 3.7964, *p* = 0.14983) as well as a sex by drug interaction (*χ*^2^ = 0.3949, *p* = 0.82083). For freezing duration, or latency to explore, we only included the 52 individuals that exhibited freezing behavior (control: *F* = 11, *M* = 10; fluoxetine: *F* = 7, *M* = 8; nicotine: *F* = 6, *M* = 10). This improved the assumptions of normality required for the ANOVA. Results of the type II ANOVA suggest that neither sex nor drug treatment have any significant effect on freezing duration (Sex: *F*_1,46_ = 1.9604, *p* = 0.1682; Drug: *F*_2,46_ = 1.3707, *p* = 0.2641). Figure 1 shows the results of freezing behaviors.

#### Speed

**Figure 2:**
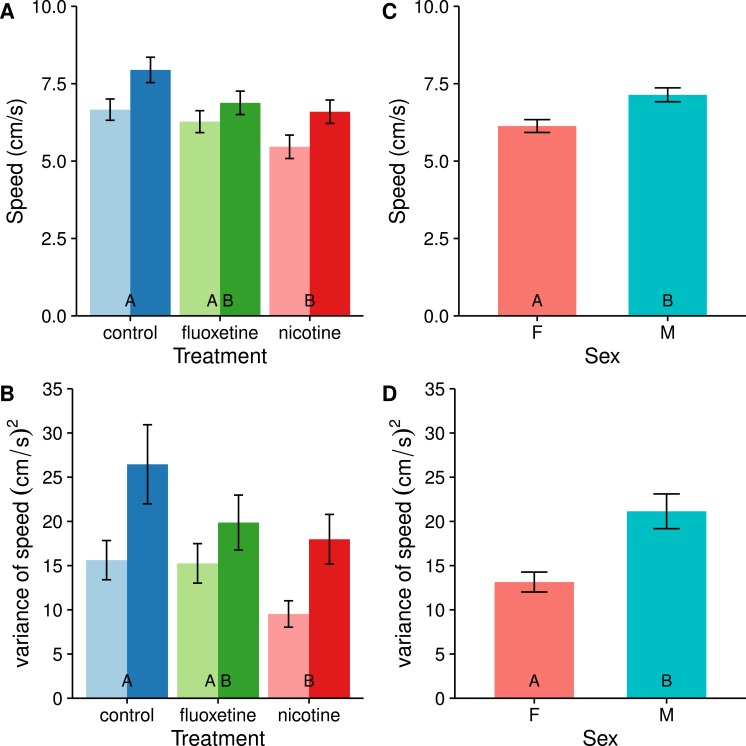
Average swimming speed (A & C) and consistency (individual variance) of swimming speed (B & D) are affected by fluoxetine and nicotine (A & B) as well as by sex (C & D). The fluoxetine treatment is not statistically different from the control, but is also not different from the nicotine treatment. Means ± SE are reported. Results of the Tukey pairwise comparisons of drug treatment groups are delineated with letter groupings where similar letters represent a non-significant difference between treatments (*p* > 0.05). In panels A & B, females are represented with light bars and males with dark bars.

When analyzing only the active swimming data from the trials, fish from both drug treatments appear to reduce their average swimming speed compared with the control, however this pattern is only significant in the nicotine treatment (*t* = 3.373, *p* = 0.0032, see Fig. 2). Drugged fish also swam at a more consistent speed than the undrugged control fish (*F*_2,81_ = 4.0654, *p* = 0.0207731), but again this trend was only significant in the nicotine treatment (*t* = 2.818, *p* = 0.0166).

#### Depth

**Figure 3:**
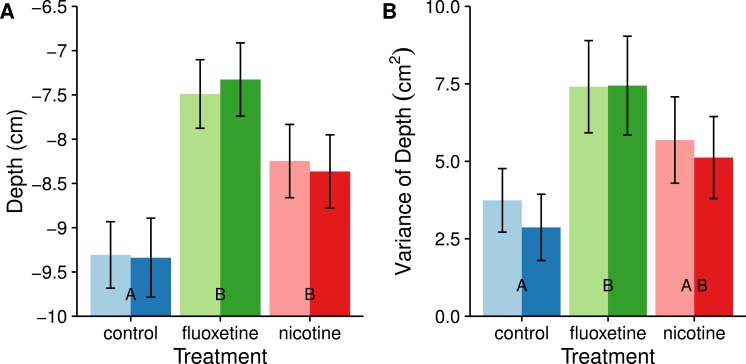
Average swimming depth (A) and average consistency (individual variance) of vertical usage (B) are affected by fluoxetine and nicotine. The nicotine treatment was not significantly different than the control with depth variance, but was also not different from the fluoxetine treatment. Means ± SE are reported and the results of the Tukey pairwise comparisons of drug treatment groups are delineated with letter groupings where similar letters represent a non-significant difference between treatments (*p* > 0.05). Sex is distinguished by females with light bars and males with dark bars.

Both the subjects dosed with nicotine and fluoxetine positioned themselves higher in the water column than the control fish (nicotine: *t* = − 2.462, *p* = 0.0417; fluoxetine: *t* = − 4.711, *p* < .0001). Fish dosed with fluoxetine explored more of the water column than control subjects (*t* = − 3.172, *p* = 0.0060). Subjects dosed with nicotine also exhibited more variation in depth use on average than the control subjects, but this difference was not significant (see Fig. 3).

**Figure 4:**
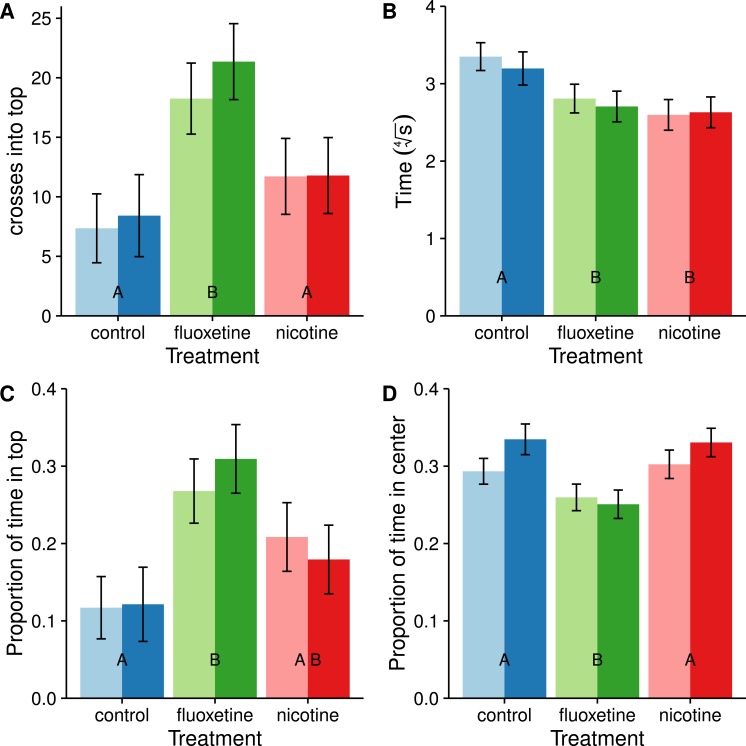
Average number of entries into the top half (A), latency to enter the top half (B), proportion of time spent in the top half (C), and proportion of time spent in center (D). Means ± SE are reported and the results of the Tukey pairwise comparisons of drug treatment groups are delineated with letter groupings where similar letters represent a non-significant difference between treatments (*p* > 0.05). Sex is distinguished by females with light bars and males with dark bars. Latency to enter the top half is transformed using a fourth root transformation in order to meet the assumption of normality in the ANOVA.

We also divided the tank into two discrete and equal vertical zones and compared the proportion of time spent in the upper half (Fig. 4). Subjects dosed with fluoxetine tended to spend more than twice as much time in the upper half as control subjects and this difference is significant (*t* = − 3.883, *p* = 0.0006). Subjects in both the nicotine and fluoxetine treatments exhibited a reduced latency time to first enter the top half than control subjects (nicotine: *t* = 3.333, *p* = 0.0037; fluoxetine: *t* = 2.652, *p* = 0.0258). When comparing the total number of visits to the top half, only the fluoxetine group showed a significant increase over the control (*t* = − 3.801, *p* = 0.0008).

#### Horizontal place preference

All subjects spent most of their time near the edges avoiding the center (Fig. 4), consistent with the concept of thigmotaxis. However, subjects dosed with fluoxetine spent less time in the center and more time near the edges than subjects in the control and nicotine treatments (*t* = 3.257, *p* = 0.0046) which is inconsistent with a reduction in thigmotaxis resulting from a reduction in stress. At this time we are unsure how these results relate to anxiolytic properties of the drug.

## Discussion

### Differences in fluoxetine and nicotine behavioral responses

Small prey fish such as zebrafish tend to behave in such a way as to reduce risk of predation. When placed in a novel open field, such behavioral strategies include diving to the bottom and remaining motionless (Egan et al., 2009), and avoiding potentially risky locations such as the surface of the water (Wilson & Godin, 2009; Oswald, Singer & Robison, 2013). Exposure to anxiolytic drugs alters these behaviors in ways that may indicate an association between anxiety related behaviors and risk management. We observed a decrease in bottom dwelling and an increase in time spent in the top half of the tank in fish exposed to fluoxetine (Figs. 3 and 4). This is consistent with patterns observed by Egan et al. (2009) who also report an increased use of the top of the water column by zebrafish exposed to fluoxetine. However, the study by Egan et al. (2009) also reports a reduction in freezing bouts and freezing time, a pattern we failed to observe. One explanation for this discrepancy could be differing effects of chronic and acute dosing. Fluoxetine is metabolized into norfluoxetine, its active metabolite, in the liver by cytochrome P450 enzymes (Rasmussen et al., 1995). It then travels through the bloodstream to the brain where it blocks the reuptake of serotonin (Beasley, Masica & Potvin, 1992). Metabolism of the drug could delay its effect until after the animal had already recovered from freezing behavior.While most fluoxetine studies utilize chronic exposure, we have shown that similar behavioral changes can occur with just a single acute dose. Acute exposure to fluoxetine has also been shown to reduce cortisol levels of zebrafish exposed to a stressful environment (De Abreu, Koakoski & Ferreira, 2014). We speculate that the behaviors we observed may be due to a reduction in physiological stress response resulting from exposure to the drugs, though more experiments are needed to confirm this.

We observed changes in swimming speed, average depth, and latency to enter the top in fish exposed to nicotine. Fish exposed to nicotine were quicker to enter the top and swam higher in the water column on average compared to control fish. This is consistent with a reduction in anxiety related behaviors as seen in the fluoxetine treatment group. Exposure to nicotine and fluoxetine appeared to decrease swimming speed while increasing the consistency at which the fish swam. The increased consistency (reduction of individual variance) might be explained by a reduction in anxiety, where individuals that are calm should move at a fairly normal and constant pace, while anxious individuals may constantly alter their swimming speeds in an erratic fashion Gerlai, Fernandes & Pereira (2009). Egan et al. (2009) reported an increase in average swim speed with exposure to fluoxetine, which contrasts with our observations of slower average swim speeds with exposure to either fluoxetine or nicotine. Sackerman et al. (2010) suggests that nicotine may have sedating effects which could account for the slower swim speeds. However, we also observe slower average swim speeds in the fluoxetine treatment, and though the difference is not statistically different from the control, it is also not different from the nicotine effect. We observed a similar pattern in the nicotine treatment with respect to the time spent at the top and the variation in depth use, where the nicotine treatment was statistically indistinguishable from both the control and the fluoxetine treatments. In these two instances, it is likely that the nicotine is having an anxiolytic effect, but that we used too low a dose to observe an effect that is different from the control. Sackerman et al. (2010) also failed to observe an effect of nicotine on swim depth using a low dose of 25 mg/L, but noted that higher doses such as 50 mg/L and 100 mg/L do produce a significant effect (Levin, Bencan & Cerutti, 2007). Our dose of 1 mg/L is noticeably lower than other studies of nicotine in adult Zebrafish, accounting for the our use of pure nicotine liquid while the other studies used a nicotine tartrate salt (Levin, Bencan & Cerutti, 2007; Sackerman et al., 2010). It should be noted that the relationship between the tartrate salt and pure form is about 0.325, such that a concentration of 100 mg/l of the tartrate equates to a concentration of 32.5 mg/l of pure nicotine (Matta et al., 2007).

Both nicotine and fluoxetine affected behavior in ways indicative of a reduction of anxiety. However, the two drugs also appear to affect different components of behavior. Nicotine had its highest effect on swimming speed, while fluoxetine mostly affected behaviors related to vertical positioning. This suggests that anxiety is not a simple condition, but rather a complex idea encompassing a number of components that are sometimes correlated, but not always connected. These behavioral components may be separated by different physiological pathways which could explain why different classes of drugs affect specific behaviors.

### The effect of sex on behavior and drug efficacy

Sex differences in anxiety behaviors have been described in a number of species including rats (Mehta, Wang & Redei, 2013), stickleback (King et al., 2013), and guppies (Harris et al., 2010). While most of these studies find that males are typically more bold (less anxious) than females, our lab has previously observed the opposite trend in the Scientific Hatcheries strain of zebrafish with regard to association with humans, vertical position, and feeding latency in individual home-tank observations (Oswald et al., 2013; Oswald, Singer & Robison, 2013; Benner et al., 2010). These differences are the basis for our inquiry as to whether substances known to alter these behaviors might work at different efficacy in males and females. In the present study, we only observe significant behavioral differences between the sexes with respect to swimming speed. While males swim slightly faster than females, it’s the females that swim at a more constant rate. In addition, males seem to show a higher probability to exhibit freezing behavior across all three treatments, and even though this trend isn’t statistically significant, it still leads us to suggest that males could be behaving with higher anxiety levels than females.

With the active swimming behaviors, we fail to observe differences between the sexes, and across all of the behaviors, the data do not suggest any indications of sex-specific effects of either drug. There is plenty of literature in mammalian models that contradict these findings (Mitic et al., 2013; Leuner, Mendolia-Loffredo & Shors, 2004; Lifschytz et al., 2006; Monleón et al., 2002; Hodes et al., 2010). One possible explanation for our lack of sex-specific effects stems from our general lack of sex differences in the behaviors analyzed, and perhaps a baseline difference in behavior is necessary to elicit a sex-specific effect. The results of Mitic et al. (2013); Leuner, Mendolia-Loffredo & Shors (2004) and Lifschytz et al. (2006) in rats all observe sex-specific responses to fluoxetine only when the sexes differed in behaviors without the drug. We do not have adequate data to confirm this explanation and more experimentation along with physiological data would be necessary.

Another possible explanation for our lack of sex-specific drug effects could be our choice of dose. Our choice of 1mg/L of nicotine is quite low compared with other studies in zebrafish (Levin, Bencan & Cerutti, 2007; Sackerman et al., 2010), and while our dosage of fluoxetine was much higher than is typically reported (Egan et al., 2009; Wong, Oxendine & Godwin, 2013), it is typically administered chronically. We would also like to note that the sex-specific results of Faraday, O’Donoghue & Grunberg (1999) utilizing nicotine in rats was only observed in one of the two strains used. Zebrafish are highly genetically diverse (Parichy, 2015) and strain differences in behavior (Benner et al., 2010; Egan et al., 2009) and drug efficacy (Sackerman et al., 2010) have been reported. Therefore the possibility exists for sex-dependent drug effects to be observed in another strain.

Finally, we cannot dismiss the possibility that zebrafish simply don’t exhibit sex-specific effects with fluoxetine or nicotine. While there is no literature in this species to compare our results with, a recently published study utilizing medaka (*Oryzias latipes*), another small teleost fish from southeast Asia, fails to find sex-specific effects of chronic fluoxetine on many of the same behaviors described in the present study (Ansai et al., 2016). More research is necessary to confirm any of the explanations given for our lack of observed sex-drug interactions. The absence of studies considering sex-specific effects of drugs is problematic if zebrafish are to remain a relevant model of pharmacology research. The topic has become a concern in all animal models that NIH is going to start requiring all animal research to include sex as part of the study unless deemed unnecessary (Clayton & Collins, 2014). If it turns out that strain is a major factor influencing our results, then the abundance of genetically diverse populations could make zebrafish an exciting tool to aid in the growing field of pharmakogenetics and personalized medicine in which genetic background, among other traits, will be important for determining what drugs will be most effective for treating disorders.

## Additional Information and Declarations

### Data Availability

The following information was supplied regarding data availability: Singer, Matthew (2016): Data for Anxiolytic effects of fluoxetine and nicotine exposure on exploratory behavior in zebrafish. figshare. https://dx.doi.org/10.6084/m9.figshare.2065407.v1.
